# Variability Modeling of Rainfall, Deforestation, and Incidence of American Tegumentary Leishmaniasis in Orán, Argentina, 1985–2007

**DOI:** 10.1155/2014/461013

**Published:** 2014-12-18

**Authors:** Juan Carlos Rosales, Hyun Mo Yang, Orlando José Avila Blas

**Affiliations:** ^1^Departamento de Matemática, Facultad de Cs. Exactas, U.N.Sa, Avenue Bolivia 5150, A4408FVY Salta, Salta Province, Argentina; ^2^EPIFISMA, Universidade Estadual de Campinas, CP 6065, 13083-859 Campinas, SP, Brazil

## Abstract

American tegumentary leishmaniasis (ATL) is a disease transmitted to humans by the female sandflies of the genus *Lutzomyia*. Several factors are involved in the disease transmission cycle. In this work only rainfall and deforestation were considered to assess the variability in the incidence of ATL. In order to reach this goal, monthly recorded data of the incidence of ATL in Orán, Salta, Argentina, were used, in the period 1985–2007. The square root of the relative incidence of ATL and the corresponding variance were formulated as time series, and these data were smoothed by moving averages of 12 and 24 months, respectively. The same procedure was applied to the rainfall data. Typical months, which are April, August, and December, were found and allowed us to describe the dynamical behavior of ATL outbreaks. These results were tested at 95% confidence level. We concluded that the variability of rainfall would not be enough to justify the epidemic outbreaks of ATL in the period 1997–2000, but it consistently explains the situation observed in the years 2002 and 2004. Deforestation activities occurred in this region could explain epidemic peaks observed in both years and also during the entire time of observation except in 2005–2007.

## 1. Introduction

Leishmaniasis is a complex of vector-borne diseases caused by protozoa of the genus* Leishmania*. The parasite is transmitted to humans through bites of female sandflies (Diptera: Psychodidae: Phlebotominae), as mentioned textually in [1]. In Argentina this disease was described for the first time in 1915 [2]. In 1928, Bernasconi described the geographical distribution of leishmaniasis in Argentina [3]; however, the first well-documented outbreak which occurred during 1984–1987 in the northeastern Salta province was described by Sosa-Estani et al. [4]. Recently, Salomón and colleagues [5] analyzed the spatial and temporal distribution patterns with regard to sandflies, transmitter of American Tegumentary Leishmaniasis (ATL) in Salta, specifically in the towns of Pichanal, Embarcación, and Mosconi (22°30′S to 24°10′S, 63°10′S to 64°25′W).

In tropical regions of America, it is believed that the occurrence of leishmaniasis transmission has been restricted to humid sylvatic regions in which humans were exposed to the parasite during forest-related activities. However, human-induced transformation of ecosystems has facilitated rapid invasion of some vectors and mammals species into nonsylvatic habitats, thereby increasing both human exposure and risk of infection. More specifically, previous studies in this region of Department of Orán provided conclusions about the potential urban transmission of ATL. By clustering studies, Gil and colleagues [6] estimated a higher probability of ATL transmission associated with children living in the vicinity of plantations and secondary vegetations.

The dynamics of the disease is correlated with fluctuations in populations of reservoirs and vectors and strongly correlated with environmental changes and climatic factors [7]. The association of ATL transmission with forest proximity/deforestation has led to the view that a large-scale landscape transformation may reduce ATL emergence [8]. Environmental changes, particularly changes in weather, are the explanations most often invoked for the occurrence of seasonality in vector-borne infectious diseases. Statistically significant correlations between epidemic cycles and cycles of temperature, humidity, rains, or winds have been identified. However, correlations may be found with confounders as well as with causes [9]. Possible effects on the increased incidence of ATL in relation to changes in the environment are caused by deforestation in order to develop agricultural projects, and, also, by urbanization progress, roads, and dams construction, as referred to in [10]. Deforestation of forests and defragmentation in Latin America are associated with an increased incidence of leishmaniasis. Moreover, the removal of vegetation can significantly alter weather patterns [11].

Qualitative and quantitative analyses that take into account the incidence of ATL in Orán were conducted in order to describe how rainfall and deforestation could influence this incidence. Since, among the factors that usually are associated with epidemic peaks of ATL, rainfall and deforestation have significant weight as described in [5, 12], they play an important role for mathematical modeling describing the complex cycle of transmission of ATL.

In this paper, the analysis of ATL incidence in Salta is limited to only two of the elements associated with environmental change: rainfall and deforestation. There are many other risk factors that influence ATL transmission in a complex way, but they are beyond the scope of this study. The set of databases that holds the currently registered ATL cases in Salta is in a one-dimensional time series. Although there are numerous research papers in which databases including satellite images of the geographical region were used for estimating the normalized difference vegetation index (NDVI) and enhanced vegetation index (EVI), such as in [13–17], in this work, the methodology is based exclusively on temporal modeling: tests for one-dimensional temporal variables are carried out. In a near future spatial data will be incorporated to complement this study incorporating NDVI and EVI, in order to have a more robust space and time mathematical modeling.

This paper presents an analysis of temporal patterns of incidence of ATL among humans in the Department of Orán (the most important town is San Ramón de la Nueva Orán, 23°08′S, 64°20′W), Salta, Argentina, for a period of 23 years. In this analysis rainfall and deforestation time series were taken into account. From the recorded incidence of ATL in this region, during the years 1985–2007, time series of the square root (sqrt) transformed variance was smoothed by moving average of 12, 16, and 24 months, respectively. The same procedure was applied to the time series of the (sqrt) transformed rainfall during the specified period. A database with the amount of hectares deforested in the Department of Orán for different length periods measured in years allowed us to construct a piecewise linear time function. Considering three time series obtained using three moving averages mentioned above, qualitative and quantitative analyses were performed in order to describe how deforestation and precipitation could influence or explain the dynamics of incidence of ATL in the Department of Orán.

Briefly, from the point of view of the dynamics of incidence of ATL, a specific contribution of this work is the finding that, in the period studied in the Department of Orán, the months April, August, and December have special features in ATL transmission. These months are associated with maximum average of the relative (sqrt) incidence that occurred in a year, arising from applying the concept of congruence. In order to show those special features, we have considered that April, August and December correspond to equivalence classes of 4, 8 and 12, respectively, and also we have taken into account that 4 ≡ 16(mod 12), 8 ≡ 2 × 16(mod 12), and 12 ≡ 3 × 16(mod 12), where 16 was obtained from the analysis of partial autocorrelation function (PACF) of the time series under studying. The observed pattern of these functions presents evidence to classify them as a process with slow decreasing, taking small values from the month 16, with the functions remaining between the bounds ±0.12. Although the use of an autoregressive model would not be suitable due to the fact that we should have a large number of parameters to estimate, any model with order 16 would be little parsimonious; hence statistical models for the periods 4, 8, and 12 could be more appropriate for the purposes of describing the behavior of the annual epidemic peaks. The dynamics of incidence of ATL in the studied period is best explained by the variability of deforestation rather than by rainfall variability. In this study it was possible to show graphically the association between epidemic peaks and deforestation, through a significant link between sets of relative (sqrt) incidence and the deforestation built for this purpose. This work opens the door for the design of mathematical models involving a larger number of variables related to climate change in the region of study.

From the epidemiological point of view, health authorities would be assisted to take necessary control measures. For example, assuming that in a given year the incidence increased in the period April–June, in the next year, the potential growth should be expected to be in the period August-September or December-January. This may be key to the anticipation of possibly increased occurrence of cases and, then, to optimize control measures by the Programa Nacional de Leishmaniasis del Ministerio de Salud Pública de la Argentina [18] and ensure more efficient use of available resources. In this sense the results help to strengthen the health structures prepared for an expected increase in ATL cases in the months of special features. Knowledge of these months or periods of higher transmission may allow the professionals in primary Health Care or trained local references to improve prevention methods included in the programme. Logically this consideration must be done taking into account the existence of a gap from the time the bite of sandfly and the onset of the first symptoms of the disease. In addition the results could help in the development of models to assess the effectiveness of different control strategies to diminish the transmission of ATL.

## 2. Materials and Methods

### 2.1. Region under Study and Incidence Data of ATL

Monthly recorded number of cases of ATL occurred in the Department of Orán, located in the west northeastern of Salta province, Argentina, during the period of the years 1985–2007, are those registered in Program of Dermatologic Diseases, in the Orán Institute for Tropical Diseases, and Hospital* Eva Perón* in Hipólito Yrigoyen. The incidence data are those confirmed by trained health workers through clinical examination of symptomatic cases of ATL. From this database, the monthly incidence per 10^5^ inhabitants was estimated. Population data were retrieved from INDEC (National Institute of Statistics and Census, Argentina) [19], in the years 1980, 1991, and 2001, and a linear regression was done in order to describe the monthly incidence of ATL.

The climate in the region under study is subtropical with a dry season encompassing a period of eight months. In summer, the high temperatures and constant humidity become uncomfortable, while during winter, the climate is comfortable due to little rainfall and low temperatures. Rainfall is around 1000 mm per year on average, with the maximum temperatures averaging 32°C in summer and a minimum of 9°C in winter. From SMN (the National Meteorology Service, Argentina) data [20], the maximum temperature recorded was 44.4°C and the minimum −3.6°C, showing a very large amplitude especially concentrated in July–September.

### 2.2. Deforestation and Rainfall Data

In order to assess the dependency of incidence of ATL with rainfall and deforestation, the deforested area (ha/year) data from 1977 to 2008 in Salta were used, in particular municipal counties of Department of Orán [21]. We constructed a linear piecewise function for monthly deforestation data for the period 1985–2007. The rainfall data were obtained also from SMN.

### 2.3. Data Processing

The following steps were carried out.

(a) Exploration of incidence, relative rates, precipitation, and deforestation data in order to identify components in the description of these time series.

(b) Study of autocorrelation function (ACF) and partial autocorrelation function (PACF) [22–24] for the time series of ATL incidence transformed by square root (sqrt): as Abeku et al. [25] did, we calculated relative incidence in order to fit morbidity data from all municipalities of the department in the same scale. The relative incidence for the month *t* (denoted by *Y*_*t*_) was calculated as $Y_{t}=\sqrt{I_{t}}/\overset{-}{I}$, where *I*_*t*_ is the incidence of cases in the month *t* and $\overset{-}{I}$ is the overall mean of the (sqrt) transformed series. The back-transformation of the incidence is thus $I_{t}={\left(Y_{t}\overset{-}{I}\right)}^{2}$. The same procedures have been applied to (sqrt) transformed rainfall time series. By doing this the two transformed series are dimensionless and we are able to compare them in order to get a statistical model to explain their behavior.

(c) Smoothing the irregular parts of time series of relative (sqrt) incidence and (sqrt) rainfall by using simple moving averages of order 2*s* + 1 aiming at a detection of trends or seasonal components: to compute the output of a simple moving average of order 2*s* + 1, the following equation was applied:

(1) $$\begin{array}{c} Y_{t}^{*}=\overset{s/2}{\underset{j=-s/2}{\sum }}a_{j}Y_{t-j};\;t=\frac{s}{2}+1,\ldots ,T-\left(\frac{s}{2}+1\right), \end{array}$$

where *a*_*j*_ = 1/*s* for *j* = 0, ±1, ±2,…, ±*s*/2 − 1 and *a*_−*j*_ = *a*_*j*_ = 1/2*s* for *j* = ±*s*/2 [22].

(d) Application of the Ljung-Box *Q*-test to the time series of relative incidence using different lag values for the ACF of the series [22]: by using the software MATLAB [26] a binary decision is used in order to test the autocorrelation among the ACF values. By filling the vector Lags with all the different lags taken in the study, the outputs are as follows.

1. *H*-Boolean decision vector: elements with *H* = 0 indicate acceptance of the null hypothesis; that is, the model fits adequately (no serial correlation at the corresponding element of lags), and elements with *H* = 1 indicate rejection of the null hypothesis. H is the same size as Lags.
2. *P* value (*P*): vector of *P* values (significance levels) at which the null hypothesis of no serial correlation at each lag in Lags is rejected.
3. *Q*-Statistics (*Q*_*s*_): vector of the values of the *Q*-Statistics for each lag in Lags.
4. Critical value (*C*_*r*_): vector of critical values with the Chi-square distribution for comparison with the corresponding elements of *Q*_*s*_.

(e) Application of the concept of congruence to the monthly data in order to find related mismatch in the partial autocorrelation function and to estimate some characteristic of the relative (sqrt) incidence time series of ATL, if any: when there is a mismatch between two PACFs, this situation can be associated with an important algebraic concept such as congruence. It can be thought of as a generalization of the relation of equality. Let *a*, *b*, and *n* be integers with *n* > 0. Then *a* is congruent to *b* modulo *n* which provides what *n* divide *a* − *b*, which is written as *a* ≡ *b*(mod⁡  *n*). The congruence class of *x* modulo *n*, denoted by [*x*], is the set of all integers that are congruent to *x* modulo *n*, as defined in [27]; that is,

(2) $$\begin{array}{c} \left[x\right]=\left\{y\in Z\;|\;y\equiv x\left(\mathrm{mod}\;n\right)\right\}=\left\{x+kn\;|\;k\in Z\right\}. \end{array}$$

(f) Obtaining the time series with 1-year and 2-year variances using moving average, given by (1), to perform the qualitative analysis of the mean outbreaks of relative (sqrt) incidence of ATL considering rainfall.

(g) Oonstruction of a simple function *f*, as defined in [15], for the deforestation to compare it with both rainfall patterns and the relative (sqrt) incidence of ATL:

(3) $$\begin{array}{c} f\left(t\right)=\underset{k=1}{\overset{N}{\sum }}d_{k}\chi_{P_{k}}\left(t\right), \end{array}$$

where *χ*_*P*_*k*__(*t*) is the characteristic function of the set *P*_*k*_ = [*a*_*k*_, *b*_*k*_], period with different length, on which data of the deforestation exist along the whole period under study. The values *d*_1_, *d*_2_,…, *d*_*N*_ are defined by *d*_*k*_ = *d*_*P*_*k*__/(*b*_*k*_ − *a*_*k*_ + 1), with *d*_*P*_*k*__ being the value of registered deforestation *P*_*k*_ in this period per deforested rainforest hectares.

The piecewise linear function *f*_*D*_(*t*) to describe the deforestation time series, obtained from the simple function *f*(*t*) given by (3), is constructed as follows: given two intervals of consecutive periods of deforestation *P*_*k*_ = [*a*_*k*_, *b*_*k*_] and *P*_*k*+1_ = [*a*_*k*+1_, *b*_*k*+1_], the end of the penultimate year of period *P*_*k*_ joins with the beginning of the first year of *P*_*k*+1_ period, and the remaining values within each interval are given by function *f*(*t*).

The scales of time series are different, and the results in the first step have essentially descriptive and qualitative characteristics and they provide conditions to make some inferences. This kind of analysis can be useful in the construction of models or submodels to describe complex systems correlating incidence and abiotic data, such as chemical and physical environment variables.

## 3. Results

**Figure 1:**
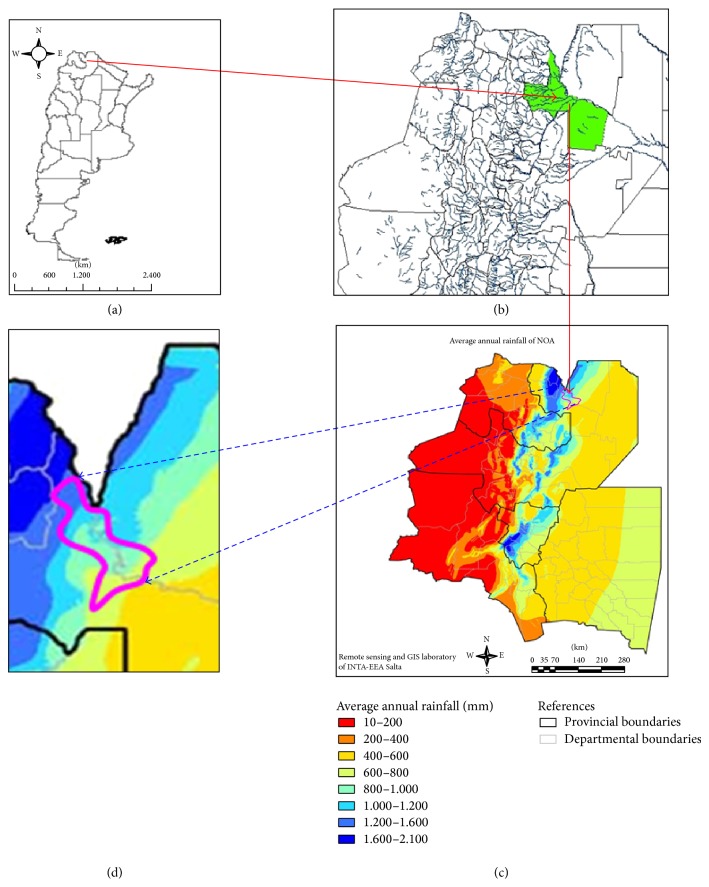
(a) Map of the Argentina Republic, the arrow starts in the province of Salta. (b) Department of Orán (green), arrow ends approximately in the region of study, taken from Instituto Geográfico Nacional (IGN) [28]. (c) Map of rainfall in the northwestern region of Argentina, taken from Instituto Nacional de Tecnología Agropecuaria (INTA) [29]. (d) Approximate region of the department of Orán which includes the cities and towns where they have registered the number of cases upon which the incidence of American Tegumentary Leishmaniasis was calculated (inside the violet curve).

Figure 1 shows the geographical location of the region of study in Argentina, and, more specifically, the west northeastern of the province of Salta, where the Department of Orán ((b) in green) is located. The map of annual precipitation for northwestern Argentina (d) and an approximate extension of the region of this study are shown ((c) inside the violet curve). Maps were obtained from Instituto Geográfico Nacional (IGN) [28] and Instituto Nacional de Tecnología Agropecuaria (INTA) [29].

**Figure 2:**
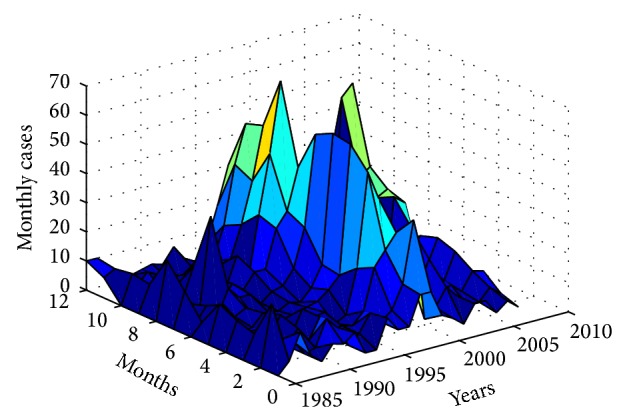
Surface based on cases of American Tegumentary Leishmaniasis in Department of Orán, Salta, Argentina. The surface shows longitudinal evolution of diseases in the twenty-three years (years) and transversal changes corresponding to monthly patterns (months).

Figure 2 shows a surface based on the monthly registered ATL cases in Orán, in the period 1985–2007. This figure shows ATL cases discriminated according to years and months. Four groups of major peaks are observed in the periods 1986-1987, 1990-1991, 1996–2000, and 2002–2004. The third period contains one subperiod (1998-1999) that presents the maximum epidemic peak registered during the period of study. In particular, in December and January, when the rainfall begins, few cases are observed with respect to the epidemic peaks.

**Figure 3:**
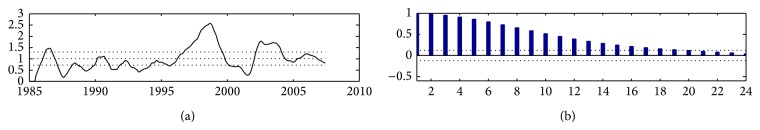
(a) Relative (sqrt) incidence of American Tegumentary Leishmaniasis cases in Orán, Salta, Argentina, period 1985–2007. (b) Sample autocorrelation function for relative (sqrt) incidence of American Tegumentary Leishmaniasis in Department of Orán, 1985–2007.

The relative (sqrt) incidence data smoothed by using moving average with lag 12, according to (1), for the period 1985–2007, are displayed in Figure 3(a). It shows a similar behavior in comparison to Figure 2. The absolute maximum represents an approximated increase of 100% (1.95) for the period 1997–1999 in comparison to 1990-1991 and 61% for the period 2002–2004. This situation keeps out of the confidence interval associated with the probable historical behavior registered until 1996. The bounds are drawn in blue and the smoothed mean series is indicated in red.

The sample autocorrelation function is shown in Figure 3(b). Based on this figure, we determinate the order of the autocorrelation component of model, which is *p* = 16. The general analytic convergence of ACF is moderated and, from results using lag 16, the values remain between the upper (+0.12) and the lower (−0.12) limits (in dashed lines) of the confidence interval indicated in Figure 3(b).

**Figure 4:**
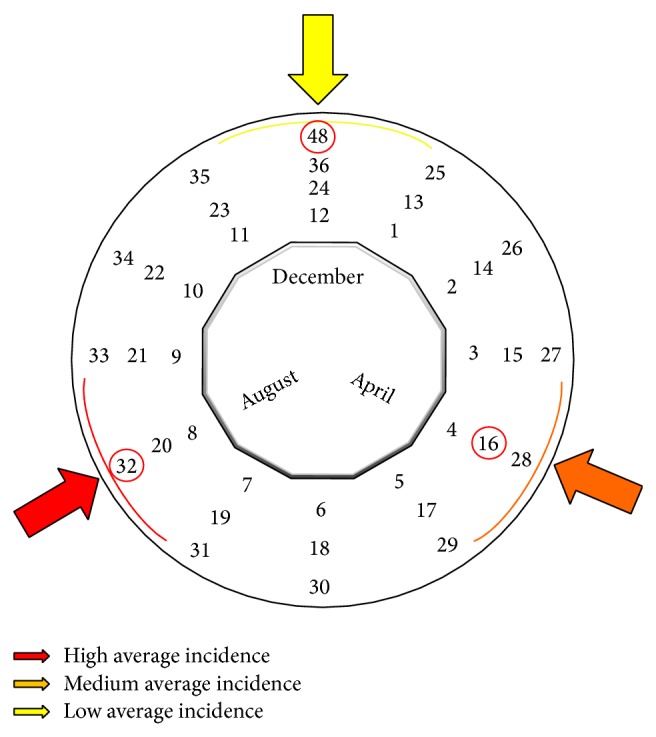
Classes of monthly relative (sqrt) incidence of American Tegumentary Leishmaniasis cases in Department of Orán, Salta, Argentina, period 1985–2007. The months April, August, and December have special features, since they are associated with maximum averages of incidence that may occur in one year, arising from the application of the concept of congruence: each month is congruent to the multiples of 16 modulo 12 respectively, where 16 was obtained from the analysis of the PACF function of the time series under study.

Based on the phase shift of 16 months found in the PACF function, it is possible to find classes of months with special features with respect to relative (sqrt) incidence of ATL. For congruence, which is defined in (2), we determine monthly classes modulo 12, for every each multiple of 16 months. The results are shown in Table 1 and Figure 4.

**Figure 5:**
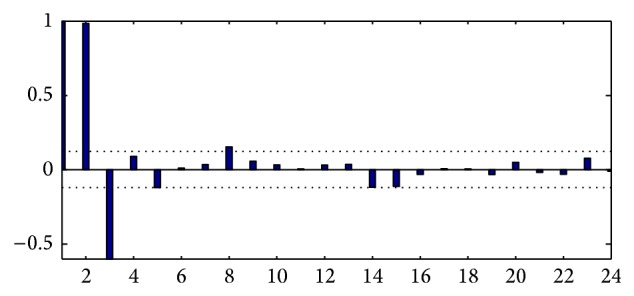
Sample PACF function of the relative (sqrt) incidence of American Tegumentary Leishmaniasis cases in Department of Orán, Salta, Argentina, period 1985–2007, smoothed with 24 months.

The partial autocorrelation function obtained for the smoothed moving average time series of the relative (sqrt) incidence with a lag of 24 months is shown in Figure 5. The figure presents a strong change in PACF values at the beginning, but after the lag of 4 months the values are situated inside the confidence bounds, so we could take on average *q* = 4 as the order of the moving average component. If we take into account a lag of 12 months, the PACF turns into an indicator of the fact that the order of moving average would be larger than 4 with a very slow convergence to zero.

We apply the Ljung-Box *Q*-test for testing goodness of fit at lags 4, 8, and 12, whose outputs come in vectors with three elements corresponding to tests at each of the three lags, respectively. The outputs are the following: *H* = [0  0  0], *P*_*v*_ = [0.790  0.984  0.999], *Q*_*s*_ = [1.703  1.898  2.004], and *C*_*r*_ = [9.488 15.507 21.026]. Due to the fact that all the components in the vector *H* are equal to 0 and the components of *P*_*v*_ are greater than the significance level (0.05), we conclude that the three null hypotheses must not be rejected; that is, in each one of the lags, the fit with moving averages is appropriated with a confidence level of 95%.

**Figure 6:**
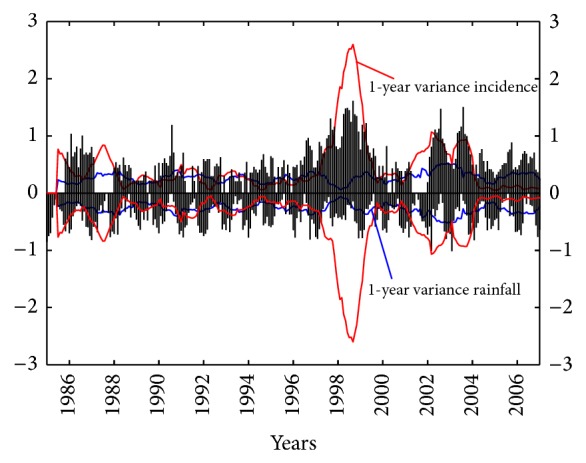
The 1-year variance time series for monthly relative (sqrt) incidence of American Tegumentary Leishmaniasis cases (red line) and (sqrt) rainfall (blue line). The relative (sqrt) incidence pattern is shown in upward axis (in black bars), while (sqrt) rainfall is shown in downward axis (in black bars) in Orán, Salta, Argentina, period 1985–2007.

Figure 6 shows the time series of 1-year variance for monthly relative (sqrt) incidence (red curve) and (sqrt) rainfall (blue curve) for the period. Both curves are presented in the same axis. The relative (sqrt) incidence pattern is shown in the upward axis (in black bars), while the (sqrt) rainfall is shown in downward axis (in black bars). By doing this, a surround effect for both silence periods and the disease epidemic peaks can be easily seen. In the period 1986–1995 there are two small peaks, and the behavior of both series is in relative agreement in the silence period. This period is followed by the most outstanding epidemic peak in which the (sqrt) rainfall variability is lower with respect to the relative (sqrt) incidence. A very short silence period then occurs in 2000-2001. In the final period there are two peaks of relative (sqrt) incidence, which coincide with two peaks of (sqrt) rainfall variability.

**Figure 7:**
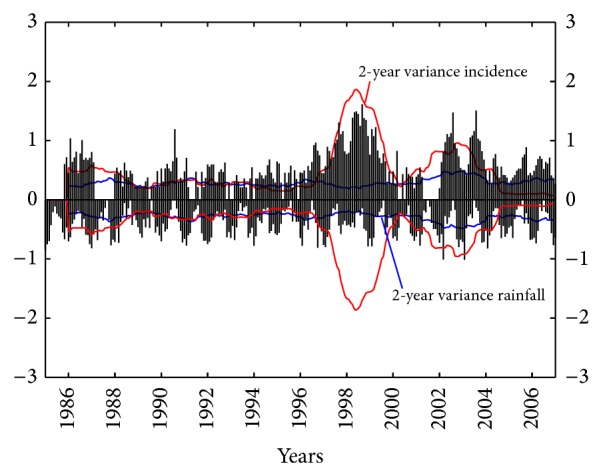
The 2-year variance time series for monthly relative (sqrt) incidence of American Tegumentary Leishmaniasis cases (red line) and rainfall (blue line) in Orán, Salta, Argentina, period 1985–2007.

Figure 7 shows the 2-year variance time series of relative (sqrt) incidence and (sqrt) rainfall. The same surround effect for both silence periods and the disease epidemic peak can be seen, as shown in Figure 6. Period 1986–1995 has a more homogeneous behavior, which is statistically significant, because the lag in series is larger with respect to the 1-year variance. The more outstanding epidemic peak presents larger variability when compared to that observed in Figure 6. A shorter silence period occurs in 2000-2001. In the final period 2002–2004 there are many peaks of relative (sqrt) incidence showing a larger variability in the relative (sqrt) incidence in comparison to the same period in the 1-year variance time series.

**Figure 8:**
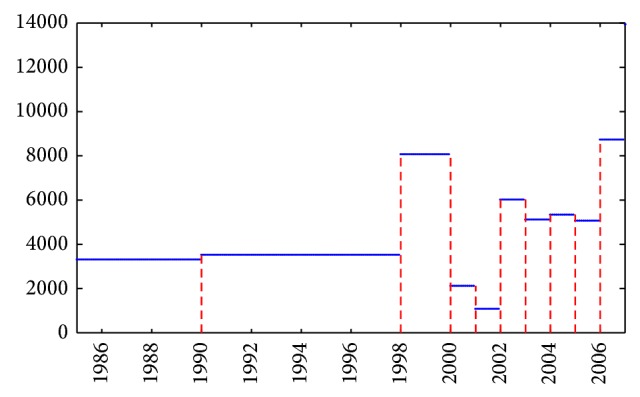
Simple function *f* for annual average deforestation, cleared hectares of forest in Department of Orán, Salta, Argentina, period 1985–2007.

Figure 8 shows the simple function defined in step (g), in which the area under the graph of *f* represents the deforested rainforest (in hectares) in the whole studied period. Between 1985 and 1998 the values of the function remained almost constant in the range 3315–3529 of hectares per year. In the period 1998-1999 the function reaches its maximum (~129%), which matches the biggest epidemic outbreak along the period of study. After these epidemics we observed a notorious decrease in the years 2000 and 2001, but from 2002 to 2005 the values of the series return approximately to the middle of the highest historical value, in which significant epidemic outbreaks are presented. This last observation could have been encouraged due to the passing by the Argentine Congress of the Law 26331 (Budget Act minimum environmental protection of Native Forest).

**Figure 9:**
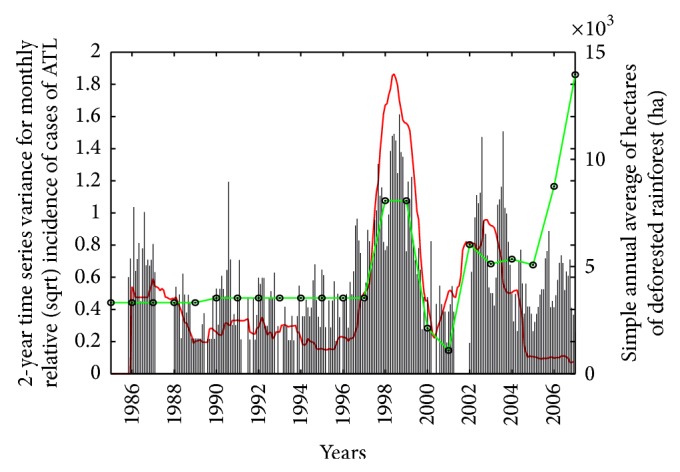
The 2-year variance times series for relative (sqrt) incidence of American Tegumentary Leishmaniasis cases (red line) and linear piecewise function *f*_*D*_(*t*) for annual average deforestation (green line) in Departament of Orán, Salta, Argentina, period 1985–2007.

Figure 9 shows the time series of 2-year variance to relative (sqrt) incidence and the piecewise linear function *f*_*D*_(*t*) (green line) constructed by joining the annual values of simple function obtained in step (g), which represents the annually deforested data in the Department of Orán. From 1986 to 1997 the values of deforested hectares were practically constant and match the homogeneous behaviour of the series under consideration. In the next period 1997–2000 a large increasing of deforested rainforest is in phase with the largest epidemic peak registered during the historical time series of incidence. In 2000-2001 the linear function has a decrease in agreement with the short silence epidemic period, while in 2002–2004 two peaks are observed showing similar dynamics with respect to the incidence. The final period presents a great particular increase of deforestation which has an inverse correlation with the incidence of ATL, and this last period presents on average the same values observed in the first period of study. A similar pattern is observed when the 1-year variance series is considered, so this figure is omitted.

## 4. Discussion

Some protozoan diseases are notifiable; that is, public health workers must inform central authority about the occurrence of these infections. In Argentina, American Tegumentary Leishmaniasis (ATL) is one of them. Although these records may contain many inaccuracies, they provide qualitative indices of longitudinal and horizontal trends in the incidence of ATL. Based on the case notification records of ATL, simple or more elaborated statistical analysis can be done, in order to look for changes in the disease incidence along time to identify seasonal periodicity. The analysis can be done rearranging data by week or month, or another suitable period of time. In this paper the construction of a surface with cases of leishmaniasis aims to visualize the epidemic situation in the Department of Orán during the period 1985–2007 (see Figure 2). The surface clearly shows when the epidemic peaks have occurred in the investigated period. Four groups of major peaks are observed in the periods 1986-1987, 1990-1991, 1996–2000, and 2002–2004. The third period contains one subperiod (1998-1999) that presents the maximum epidemic peak registered along the whole period. In particular in December and January, when rainfall begins, relatively few cases are observed in comparison to the disease peaks.

Taking into account these data we constructed smoothed time series of the relative (sqrt) incidence, Figure 3(a). From this figure and Figure 2, four groups of epidemic peaks are observed, but three of them are outside the confidence bounds, and they are statistically significant, except in the period 1990-1991, when its maximum is very close to the upper confidence bound. In these groups we observed that there are two main moments per year: autumn and spring, and the second, summer time. Taking into account the first systematic evaluation of the spatial and seasonal abundance of phlebotomine sandflies done by Salomón et al. (2004) [5] in Salta, Argentina, at a Pichanal rural peridomestic site, the epidemic peaks described here are almost in agreement with those peaks per year discovered by them. Looking at the maximum of abundance of* Lutzomyia neivai* density obtained by Quintana et al. (2012) [30] and the incidence of human cases, particularized in the same period of time, we can see a gap between them. In this way we consider that the evolution of the relative (sqrt) incidence, directly linked with the number of cases, can be seen positively associated with the abundance of phlebotomine sandflies.

Based on the relative (sqrt) incidence time series we obtained the autocorrelation function; see Figure 3(b). The observed pattern presents evidence to classify it as a process with slow decreasing, taking small values from the month 16, with the function remaining between the bounds ±0.12. The use of an autoregressive model would not be suitable due to the fact that we could have a large number of parameters to estimate; hence any model with order 16 would be little parsimonious.

We found a phase shift of 16 months, which allowed us to find classes of months with special features with respect to smoothed relative incidence of ATL. For congruence, we determined monthly classes modulo 12 belonging to multiples of 16 months, which allowed us to define monthly classes corresponding to these months April, August, and December, as shown in Table 1. In these months the relative incidence should be expected to increase according to historical records analyzed in this work. It is remarkable that these monthly classes that begin at each one of the three seasonal periods match the mean disease peaks and also the seasonal abundance of phlebotomine sandflies described by Salomón et al. (2004) [5].

The PACF function presents a more pronounced decrease, showing that after 4 months all data remained inside the confidence bounds; see Figure 5. The value at lag 8 keeps a little out, although it represents 4% of the total number of data (less than 5% significance level). It is noteworthy that, after this value, the function remained within the confidence bounds, and oscillatory behavior occurred. This specific observation would allow us to initiate a study of statistical modeling processes of the time series under study using components autoregressive and moving average, for example with an AR (4) or with a higher-order structure, preserving the parsimony of the model and other characteristics of relevance.

By applying the Ljung-Box *Q*-test for autocorrelation at lags 4, 8, and 12, all outputs came in vectors with three elements, corresponding to tests at each of the three lags. The output *H*_*i*_ = 0 means the acceptance of the null hypothesis. The vector *P* contains the *P* values for the three tests. At the *α* = 0.05 significance level, the null hypothesis of no autocorrelation is accepted in all the three lags. The conclusion is that there is significant partial autocorrelation in the series. The statistics test and critical *χ* ^2^ values are given in outputs *Q*_*s*_ and *C*_*r*_, respectively, with complementary information in order to accept or not the null hypothesis.

Salomón et al. (2004, 2012) [5, 31] and Chaves et al. (2008) [8] suggested that climate phenomenon of the rainfall is associated with water balance and runoff coefficient, shadow-roofed protection available, and relative humidity and temperature. These effects modulated by the precipitation and temperature would have in turn impact on the larval substrate, where a* window* of moisture of the soil is necessary for the survival, whose effects are different from that induced by weather variables, such as the metabolic velocity modulators on larvae but also on adults. Quintana et al. (2012) [30] discussed, from a focal level of scale changes, the landscape modifications, deforestation, and fragmentation in hyperendemic areas of northwestern Argentina, concluding that the longitudinal capture for interepidemic periods suggests a metapopulation structure dynamics for sandflies. From a time series analysis, the populations in patches of vegetation near homes have a significative correlation every 5 or 6 weeks. Such observed dynamic is almost similar to the one that we found in this paper for the relative (sqrt) incidence to human cases.

With regard to the results obtained here (see Figures 6 to 9), where only the variability of rainfall and deforestation in the region were considered, we found an important observation about the epidemic situations: first of all the time series of rainfall variance could not explain completely the epidemics in the period 1997–1999, but rainfall variability could explain the epidemics in the periods 1986-1987 and 2002–2004, specially in the second period. In a review article [30], the ATL vector showed a positive association with rainfall occurred in the previous years. Due to the generation of new eventual breeding sites, the sandflies also showed an association with the temperature and relative humidity. A better approximation of our finding to those results would be obtained by adding other variables such as temperature and relative humidity.

Figure 8 showed simple function for annual average deforestation, cleared hectares of forest in the Department of Orán, Salta, Argentina, in the period 1985–2007. From the simple function of annual mean values of deforested hectares of forest in the Department Orán, we constructed a piecewise linear function *f*_*D*_ taking only the annual values in order to compare it with the variance time series of the relative (sqrt) incidence of ATL, shown in Figure 9 (green line). When it is compared for descriptive purposes of the relative (sqrt) incidence, it presents a significative coupling with the historical ATL epidemic peaks mentioned before. This could explain the relationship of deforestation with the evidenced relative (sqrt) incidence of ATL in this study and matched the association between outbreaks and forest clearance described by Chaves et al. [8]. This figure shows that the increase in the deforestation is in phase with the main historical ATL peaks in the Department of Orán, recorded in the periods 1997-1998 and 2002–2004. With respect to the first epidemic period, we observe, in comparison with Figure 2 in the paper of Paruelo et al. (2011), that the dynamical behavior of deforested surface is almost linear with a slow slope in the period 1980–1996 and since then its patterns may be fitted by a polynomial function with higher order or perhaps with an exponential function. In particular we see in Figure 9 that from the last peaks of 2004 the increasing in the deforestation goes against the decreasing in the relative (sqrt) incidence. One possible explanation for this phenomenon may be a sudden change in the normal environment for developing the population of vector-borne disease.

On the other side we show that deforestation events did not occur with dominant frequencies and presented with low frequencies with approximate probability between 1/23 and 2/23, because piecewise linear function shows that the maximum deforested hectares per year happened once or twice up to 2005 in the studied period. In this way, the situation presented due to the deforestation is similar to the behavior described by Halley (1996) [32]: the ecologists expect both rare and common events to be important and the ecosystems will be influenced by numerous small changes each day. Some rare events will have longer-lasting influence and 1/*f*-noise is a way of describing these kinds of events. It has been described as* an evolutionary random process* and is characterized by strong correlations on a multitude of scales. This suggests that the epidemic peaks can be explained by the association of quasirare events of indiscriminate deforestation. We understand that deforestation is different in rainy or dry seasons; however, it has been chosen to build piecewise continuous function for simplicity and aims to visualize graphically the situation.

The results of this study showed that the dynamics of the relative (sqrt) incidence is strongly associated with deforestation variable and in a minor extent with variable rainfall in the period analyzed. The analysis of historical time series allowed us to observe links between incidence, deforestation, and rain and find periods of special features (April–June, August-September, and December-January), which may be key to the anticipation of increased occurrence of cases and, hence, to optimize control measures by the Programa Nacional de Leishmaniasis del Ministerio de Salud Pública de la Argentina, which was passed by Resolution 386/2004. The mentioned Programme tends to ensure more efficient use of available resources. In this sense the results help to strengthen the health structures to be prepared for an expected increase in case mix, in the months of special features. Knowledge of these months or periods indicated in Table 1 and Figure 4, at community level by professionals in primary health care and trained local personals, allows health authorities to anticipate and to improve prevention methods included in the programme.

## 5. Conclusion

The quantitative and qualitative descriptions presented here can be useful when constructing models for the purpose of predicting the temporal dynamics of ATL. For example, we showed that, from an algebraic point of view using the concept of congruence, three typical months, April (class 4), August (class 8), and December (class 12), were found and they match the mathematical description of the ACF and PACF. By studying relative (sqrt) incidence, we observed that the annual peaks of ATL begin in these months, showing that in August occurred the maximum frequency (greatest dry season). With respect to April and December, they showed lower frequencies than August. These classes were obtained using the application of congruence 16 modulo 12, where 16 was obtained from the analysis of the ACF and PACF of the relative (sqrt) incidence time series. In this study it was possible to show graphically the association among epidemic peaks, rainfall, and deforestation. A significant link between sets of relative (sqrt) incidence and the deforestation built for this purpose was observed, which is more significant than the coupling shown in the series of rainfall.

Best descriptions can be achieved by incorporating other climatic factors, in particular time series presenting seasonal components, such as temperature. It would be of interest to model these factors using autoregressive integrated moving average (ARIMA) models, particularly moving average (MA) models as done by Chaves and Pascual (2006) [33] and Salomón et al. (2004) [5]. A model with order 4, 8, or 12 may be suitable. However, there are difficulties to understand the effects of climatic and deforestation phenomena measured in different scales in ATL incidence, as shown in this paper. However, the use of relative incidence transformations would allow us to overcome these difficulties.

The simple descriptions obtained in this work are reasonable and our analysis can provide useful clues about understanding the nature of the underlying epidemiological processes that should be incorporated or complemented in other mathematical models for ATL (see Gonzáles et al. (2010) [7]), or another vector borne diseases (see Gomez-Elipe et al. (2007) [34]). These models can take into account different scales in time and space as well as the frameworks where the ATL transmission is suspected.

These results could help to strengthen the health structures prepared for an expected increase in case mix, specially in the typical months. Knowledge of these months or periods may allow education professionals in primary health care to anticipate and to improve prevention methods included in the Programa Nacional de Leishmaniasis del Ministerio de Salud Pública de la Argentina.

## Figures and Tables

**Table 1:** First multiple of 16 months and their respective congruence classes.

Months	16	32	48	64	80	96	⋯
Classes of months	[4]	[8]	[12]	[4]	[8]	[12]	⋯
Month of the year	April	August	December	April	August	December	⋯
